# Hashimoto’s thyroiditis-related myopathy in a patient with SARS-CoV-2 infection: A case report and systematic literature review

**DOI:** 10.1097/MD.0000000000035720

**Published:** 2023-10-20

**Authors:** Zheng Cong Lee, Yu Jun Eugene Wong, Lian Lian Ti, Digambarrao Pande Shrikant, Tunn Lin Tay, Anindita Santosa

**Affiliations:** a Department of Medicine (Rheumatology Service), Changi General Hospital, Singapore; b Department of Gastroenterology, Changi General Hospital, Singapore; c St Andrews Community Hospital, Singapore; d Department of Rehabilitative Medicine, Changi General Hospital, Singapore; e Department of Endocrinology, Changi General Hospital, Singapore.

**Keywords:** case report, COVID-19, creatine kinase, Hashimoto’s thyroiditis, myopathy

## Abstract

**Rationale:**

Hashimoto’s thyroiditis (HT) is a common autoimmune disease. However, its presentation and management in the context of COVID-19 are unclear, and COVID-19-triggered HT, along with myopathy and persistent creatine kinase (CK) levels, have not been previously reported. Moreover, no literature review is currently available on HT in the context of COVID-19. This study is a case report and systematic review of the literature.

**Patient concerns:**

A 33-year-old man was admitted with acute-onset myalgia, anosmia, loss of taste, fever, and upper respiratory tract symptoms.

**Diagnoses:**

He was diagnosed with coronavirus disease (COVID-19) during hospitalization and had abnormal CK levels. The elevated CK level persisted even after the resolution of COVID-19. After excluding myopathies and cardiac factors, HT was diagnosed.

**Interventions:**

CK levels did not decrease appreciably until 14 d after levothyroxine administration.

**Outcomes:**

The patient was discharged from the hospital in good health. In the systematic literature review, 7 case reports on COVID-19-associated HT were observed, although no incidence of associated myopathy or persistent elevation of CK was noted.

**Lessons:**

This case report highlights the potential link between COVID-19 and autoimmune thyroid diseases. In particular, this study underscores the significance of recognizing new-onset autoimmune thyroid disease in COVID-19-positive patients with elevated CK levels that cannot be attributed to other factors. This systematic review offers additional perspectives for diagnosing and managing HT in COVID-19 settings. Overall, the findings of this study could have important clinical implications for the care of COVID-19 patients, as early identification and treatment of autoimmune thyroid disease could help prevent long-term complications. Additional research is essential to elucidate the fundamental correlations between COVID-19 and HT and assess the effectiveness of therapeutic approaches for autoimmune thyroid conditions related to COVID-19.

## 1. Introduction

Severe acute respiratory syndrome coronavirus 2 (SARS-CoV-2) infection can aggravate existing autoimmune conditions and precipitate severe immune-mediated diseases, which pose significant morbidity and mortality risks.^[1]^ Hashimoto’s thyroiditis (HT) is a common autoimmune disease frequently presenting with neuromuscular symptoms. This T-cell-mediated condition is characterized by elevated serum levels of antithyroid peroxidase antibodies.^[2]^ HT can result in short- and long-term complications. Patients may experience short-term fatigue, weight gain, dry skin, and constipation. If left untreated or poorly managed, HT can cause long-term complications, including hypothyroidism, goiter, and an increased risk of thyroid cancer.^[3]^

Furthermore, some patients develop additional autoimmune disorders. Consequently, timely and appropriate treatment is vital for preventing these complications. Viral infections such as COVID-19 are also linked to myalgia and myopathy.^[4]^ Myalgia is among the most commonly reported symptoms in COVID-19 patients, often preceding fever and other manifestations.^[4,5]^ However, to our knowledge, no cases of COVID-19-induced HT accompanied by myopathy have been reported. Moreover, a comprehensive review focusing on COVID-19-induced HT is lacking.

Herein, we describe a rare clinical case of myopathy and Hashimoto’s thyroiditis (HT), which was unmasked by COVID-19 infection. This study also encompasses a systematic review of the existing literature along with an in-depth discussion of the putative mechanistic basis of HT in the context of COVID-19.

## 2. Case report

A 33-year-old Bangladeshi man was admitted to the hospital with a two-day history of generalized myalgia, subjective weakness in both legs, anosmia, and loss of taste. He subsequently developed upper respiratory tract symptoms on the third day and fever on the tenth day. Polymerase Chain Reaction (PCR) results from a nasopharyngeal swab confirmed the preliminary diagnosis of COVID-19.

Upon admission, the patient reported no orthopnea, lower-extremity swelling, dyspnea, chest discomfort, palpitations, or orthopnea. Other system assessments were unremarkable. The patient was normotensive and non-tachycardic, with a normal cardiorespiratory examination. The neurological examination findings were within the normal range, and no muscle pain or rashes were observed. Abnormal laboratory results included significantly elevated creatine kinase (CK) levels (5358 U/L; Table 1).

**Table 1:** Overview of crucial laboratory results.

Parameter	Units	Value	Reference range
CK	U/L	5358	40–210
CKMB	ng/mL	20.10	<5.00
Troponin T, serum	ng/L	42	0–29
Aldolase	U/L	21.7	1.3–6.3
Free T4	pmol/L	<5.50	10.00–20.00
TSH	mIU/L	216	0.400–4.00
Anti-TPO	IU/mL	78.53	<5.50
Antinuclear antibody, serum		1:160	<1:80 (Homogenous pattern – IIF)
Anti-ENA profile (anti-Ro, anti-La, anti-Sm/RNP, anti-Scl70, anti-Jo-1)		Negative	
Anti-dsDNA	IU/L	<20	<20
Myositis Profile - EUROIMMUNE Euroline Immunoblot, Autoimmune Inflammatory Myopathies 16-Antigens		Negative	Non-reactive
Anti-HMGCR		Negative	Non-reactive

Aldolase = Aldolase enzyme, Anti-dsDNA = Anti-double stranded DNA, Anti-ENA = Anti-extractable nuclear antigens, Anti-HMGCR = Anti-3-hydroxy-3-methylglutaryl-coenzyme A reductase, Anti-Jo-1 = Anti-histidyl-tRNA synthetase, Anti-La = Anti-La/SS-B, Anti-Ro = Anti-Ro/SS-A, Anti-Scl70 = Anti-topoisomerase I, Anti-Sm/RNP = Anti-Smith/Ribonucleoprotein, Anti-TPO = Antithyroid peroxidase, CK = Creatine kinase, CKMB = Creatine kinase-MB, Free T4 = Free thyroxine, Homogenous pattern (IIF) = Indirect immunofluorescence test, Myositis Profile = Autoimmune inflammatory myopathies 16-antigens, Troponin T = Troponin T, serum, TSH = thyroid-stimulating hormone.

Electromyography revealed a mild diffuse myopathic process with patchy areas of irritability in the gastrocnemius medial muscle but no evidence of large fiber polyneuropathy. Transthoracic echocardiography revealed a non-dilated left ventricle, impaired systolic function, and a left ventricular ejection fraction of 46%, with mild hypokinesia and no valvular lesions.

Elevated CK levels were initially attributed to COVID-19-related myopathies. Intravenous hydration was initiated, leading to an initial decline in CK levels. On day 9, the patient experienced severe dyspnea with 91% desaturation and bilateral infiltrates in the lower lung lobes, consistent with SARS-CoV-2 pneumonitis. The patient showed improvement after 24 h of oxygen supplementation and did not require further COVID-19-specific treatment.

**Figure 1.**
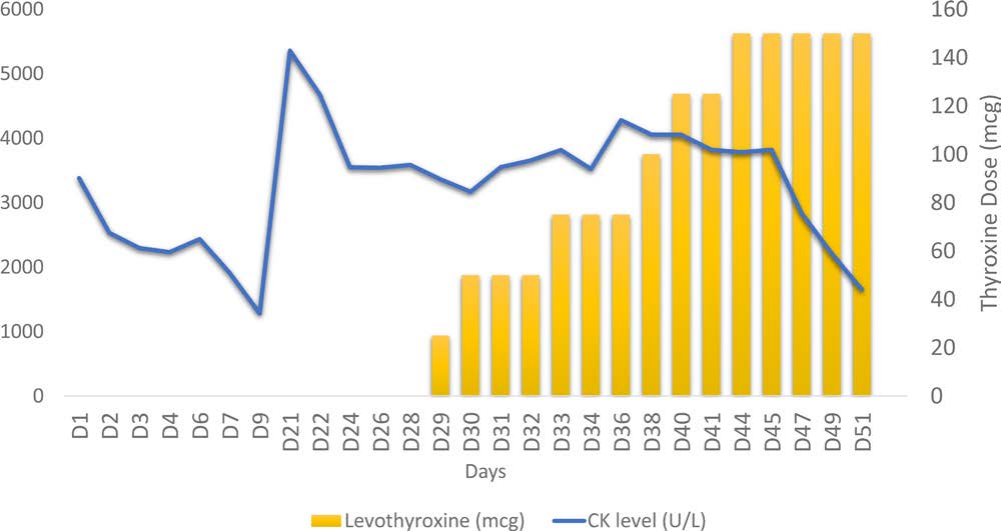
The patient’s clinical course, including CK levels and levothyroxine therapy. CK = creatine kinase.

On day 10 of admission, the patient’s CK level remained elevated (Fig. 1). Further evaluation revealed HT and probable hypothyroidism-related myopathy. The patient’s free T4 level was lower than the reference range, with a value of less than 5.50 pmol/L (normal: 10.00–20.00 pmol/L, Table 1). Thyroid-stimulating hormone (TSH) levels were markedly increased to 216 mIU/L, well above the reference range of 0.400 to 4.00 mIU/L. The patient’s anti-TPO levels were elevated to 78.53 IU/mL, compared to the normal value of less than 5.50 IU/mL. The antinuclear antibody titer was 1:160 with a homogeneous pattern. In contrast, the reference value for a homogeneous pattern was less than 1:80. However, the anti-ENA, anti-dsDNA, myositis, and anti-HMGCR test results were all negative or within the normal reference values.

Given that his CK level was exceptionally high in hypothyroidism and because of the unique electromyographic findings of irritable myopathy, we further evaluated his myocardial function and searched for other potential causes of CK increase, such as immune-mediated myopathy. On admission days 22 and 23, subsequent nasopharyngeal swabs tested negative for SARS-CoV-2. Levothyroxine replacement therapy was initiated on day 29 of admission (starting at 25 µg per day) and was gradually titrated to 150 µg per day. CK levels decreased by the end of the third week following thyroxine treatment initiation.

Figure 1 shows the patient’s clinical course. The information was broken down by the day of admission, days from the onset of COVID symptoms, creatine kinase (CK) levels, hydration status, COVID PCR test results, and levothyroxine dosage. The patient’s CK levels fluctuated throughout their stay, with the highest level of 5358 U/L on day 21 and the lowest level of 1650 U/L on day 51. The hydration status shows when the patient started, continued, or stopped hydration treatment, while the COVID PCR test results indicated that the patient tested positive initially but then negative on days 24 and 25. Lastly, the Levothyroxine dosage section shows the progression of the patient’s levothyroxine treatment, with an increase in dosage from 25 to 150 µg during their hospital stay. CK levels improved at the end of the third week after initiating levothyroxine treatment.

As the patient’s CK level did not decrease after hydration and thyroxine supplementation over the next 2 weeks, our next strategy was to schedule magnetic resonance imaging of the affected muscles or a muscle biopsy for myopathy examination. However, treatment was postponed due to budgetary considerations, and the patient remained asymptomatic.

### 2.1. Systematic review

#### 2.1.1. Search strategy.

Data for this case-based review were obtained through a systematic and comprehensive literature search using the following keywords: “COVID-19” AND “myositis,” “COVID-19” AND “rhabdomyolysis,” “COVID-19” AND “myalgia” to identify articles from PubMed and Google Scholar without language restrictions. We also performed a secondary search on.” “hypothyroidism” AND “myositis.” Search strings for “Hashimoto’s thyroiditis” AND “COVID-19,” “myositis” AND “COVID-19” AND “hypothyroidism,” “myositis” AND “COVID-19” AND “Hashimoto’s thyroiditis” yielded no results. Individual case reports case series, editorials, reviews, case-control studies, and cohort studies were also examined. Duplicate publications were not considered in further analysis. The reference lists of all articles meeting the criteria and references within pertinent review articles were thoroughly reviewed to identify studies that may not have been identified in the database search. Studies reporting vaccine-induced HT were excluded from this review. A literature search was performed on February 21, 2023.

The variables extracted from the literature for our analysis included the following: study reference, year, country, sex, Age, COVID Severity, time since COVID-19, hospitalization for COVID-19, previous thyroid disease, thyroid function test (TFT), Myopathy, CK Levels, Management, and Outcome.

#### 2.1.2. Results of the systematic review.

**Figure 2.**
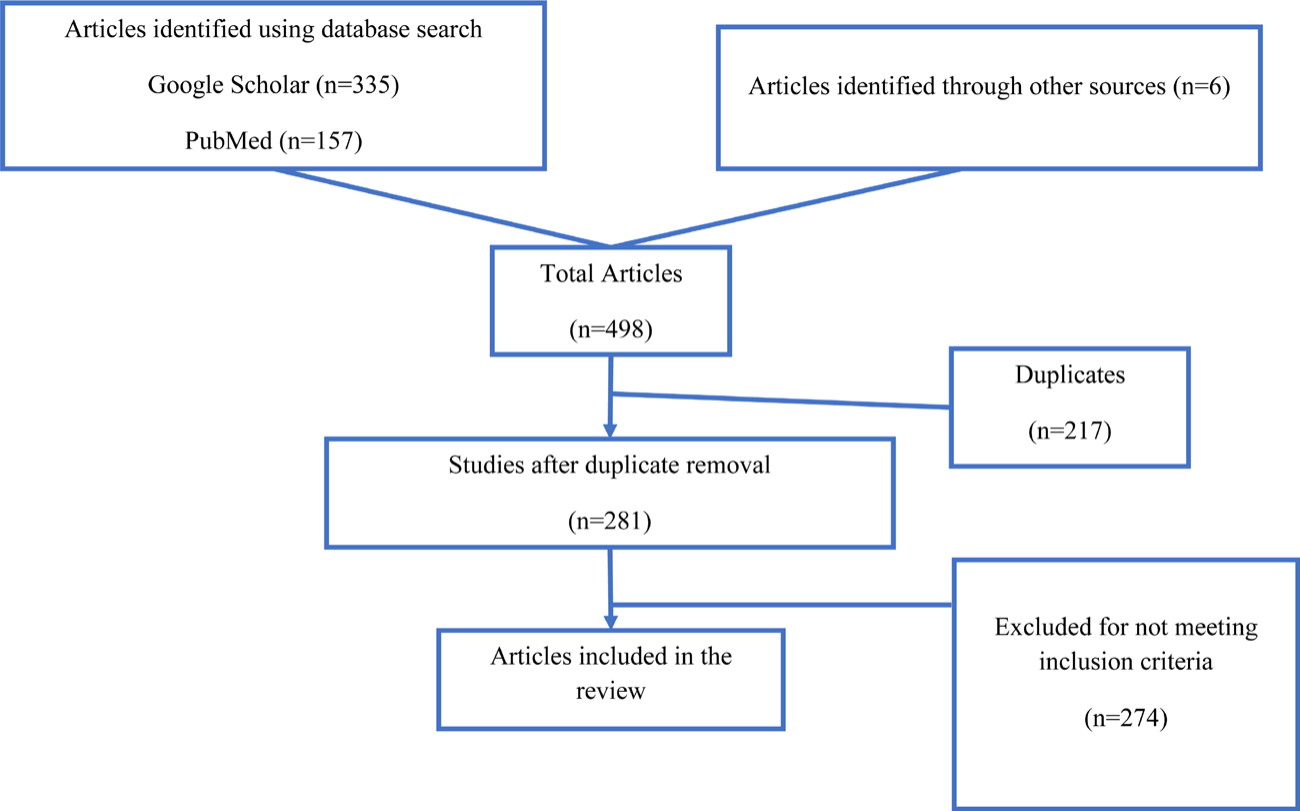
PRISMA flowchart illustrating the study selection process. PRISMA = Preferred Reporting Items for Systematic Reviews and Meta-Analyses.

The search on Google Scholar resulted in 335 hits, whereas PubMed yielded 157 hits. Screening the references of the selected articles led to the identification of 6 additional potential articles. After excluding duplicates and articles that did not meet the inclusion criteria, 7 case reports on COVID-19-associated HT were identified (Fig. 2). Our case report enriches the existing body of research by increasing the total number of documented cases on this topic to 8 and highlighting distinctive clinical characteristics and diagnostic complexities of HT in COVID-19.

Table 2 summarizes case reports of COVID-19-triggered hypothyroidism in various countries between 2020 and 2023. The patients’ ages ranged from 33 to 69 years, and most of them were female. The severity of COVID-19 varies, with some patients being asymptomatic, while others experience mild or severe symptoms. In most cases, the patients did not have a history of thyroid disease, and their TFT showed abnormalities, such as elevated TSH, low T4, and high anti-TPO levels. Myopathy has been reported in some cases, with CK levels ranging from normal to significantly elevated. The primary treatment for these patients was levothyroxine, and the outcomes were generally positive, with patients achieving euthyroidism within 1 to 4 months of treatment initiation.

**Table 2:** Synthesis of major findings from case reports on COVID-19-triggered Hashimoto’s thyroiditis (HT).

STY	YR	CTR	Sex	Age	CVDS	TCD	HOSP	Prev. TD	TFT	MYP	CK	TX	Outcome
This study	2023	Singapore	M	33	Moderate	14	Yes	No	TSH: 216 mIU/L, T4: <5.50 pmol/L, TPO: 78.53 IU/mL	Yes	5358 IU/L	Levothyroxine	The patient responded well to the treatment
Khaja, Qureshi et al 2022^[9]^	2022	USA	F	34	Asymptomatic	0	No	No	TSH: 47.10 mIU/L, T3: 34 ng/dL, T4: 0.15 μg/dL	No	1154 IU/L	Levothyroxine	The patient responded well to the treatment
Das, Ali et al 2022^[6]^	2022	India	M	33	Severe	14	Yes	NR	TPO: 159 IU/mL	No	NR	Deflazacort 5 mg + psychiatric medication	The patient responded well to the treatment
Tee, Harjanto et al 2021^[7]^	2021	Singapore	M	45	Mild	7	No	No	TSH: 6.49 IU/mL, T4: 9.19 pmol/L, TPO levels > 2000 IU/mL	No	Normal	Levothyroxine	The patient responded well to the treatment
Knack, Hanada et al 2021^[36]^	2021	Brazil	F	33	NR	20	No	No	TSH: 8 mIU/mL, free T4: 0.5 ng/dL, anti-TPO: 115 IU/mL	No	NR	Levothyroxine	Euthyroidism was achieved 4 months later
Feghali, Atallah et al 2021^[37]^	2021	USA	F	38	Mild	42	No	No	TSH: 136 mIU/L, free T4: 0.2 ng/dL, anti-TPO > 900 IU/mL, anti-thyroglobulin antibodies > 1000 IU/mL	No	NR	Levothyroxine	Euthyroidism was achieved 1 month later
Allam, El-Zawawy et al 2021^[38]^	2021	Egypt	F	42	Mild	8	No	HT for 10 years	TSH: 2.1 mIU/mL, FT4: 1.46 ng/dL, FT3: 3.46 pg/mL	No	NR	Levothyroxine	Euthyroidism was achieved 2 months later
Dixit, Truong et al 2020^[8]^	2020	USA	F	69	NR	0	Yes	NR	TSH: 61.3 μU/mL, fT4: 0.2 ng/dL, TPO: 33.4	No	1908 IU/L		N

Anti-TPO = Antithyroid peroxidase, CK Lvls = Creatine Kinase (CK) Levels, CTR = Country, CVDS = COVID-19 Severity, Free T4 = Free thyroxine, GDR = Gender/ Sex, HOSP = Hospitalization for COVID-19, HT = Hashimoto’s thyroiditis, MYP = Myopathy, NR = Not reported, Outcome = Study Outcome/ Research Findings, Prev. TD = Previous Thyroid Disease, STY = Study, TCD = Time Since COVID-19 Diagnosis, TFT = Thyroid Function Test, TSH = Thyroid-stimulating hormone, TX = Treatment/Intervention, YR = Study Year/ Data Collection Year.

Of the identified cases, 37.5% (3 of 8) were men,^[6,7]^ with most reporting no prior history of thyroid disorders. The onset time of HT ranged from concurrent with COVID-19 infection (day 0)^[8,9]^ to several days later (range 7–42 days). Only one severe COVID-19 has been reported,^[6]^ and there was no myopathy or persistent elevation of CK levels.

## 3. Discussion

Viral infections have been associated with myalgia and myopathy, and in COVID-19-positive patients, the prevalence of myalgia and myopathy is approximately 11% to 44%.^[4,5,10–14]^ However, no previous study has reported myopathy, persistent CK levels, and HT in a COVID-19-positive patient. This study, combined with a comprehensive analysis of published case reports, contributes to a better understanding of the etiology, symptoms, and management of COVID-19 patients with HT. The most significant finding of this study is the urgent need for healthcare professionals to have a heightened clinical suspicion for new-onset autoimmune thyroid disease in COVID-19-positive individuals with elevated CK levels that cannot be explained by other factors. Furthermore, this study demonstrated the effectiveness of L-thyroxine treatment in achieving euthyroidism in such patients, indicating a potential treatment option for this subset of patients. This finding has significant implications for managing COVID-19 patients and underscores the importance of investigating potential autoimmune disorders in those presenting with unexplained muscle weakness and elevated CK levels.

Rhabdomyolysis or myopathy, followed by significant elevations in CK levels, has been reported in COVID-19-positive patients.^[11,14–17]^ It is important to note that in COVID-19 patients, myalgia and muscle weakness may develop before fever and breathing problems.^[12–16,18]^ Our patient’s symptoms and early clinical development are similar to the findings of COVID-19 patients who presented with myalgia before fever onset and subsequent deterioration in respiratory function.^[5,10,12,14–16,18]^ It is worth noting that although our patient experienced persistent CK elevation associated with muscle involvement, most COVID-19 patients with similar symptoms did not have sustained CK elevation. In fact, many individuals with severe muscle tenderness and subjective weakness on admission recover quickly with supportive measures and aggressive hydration within 7 to 10 days.^[14,16–18]^ Our patient’s preexisting autoimmune condition, HT, may have contributed to persistent CK elevation and delayed recovery from muscle involvement. However, further research is needed to fully understand the underlying mechanisms and potential long-term implications of COVID-19 in patients with preexisting autoimmune conditions.

In the context of COVID-19-associated HT, Khaja et al (CK level: 1154 IU/L) and Dixit et al (CK level: 1908 IU/L) reported elevated CK levels.^[8,9]^ However, the outcome in our patient was distinct from that in these cases, showing a rebound spike in CK levels on day 21 of admission. The persistent elevation of CK levels, even after the clearance of SARS-CoV2, forced us to investigate other potential etiologies. An immunological epiphenomenon was proposed, resulting in a delayed onset of immune-mediated inflammatory myopathy, particularly irritable myopathy, based on electromyography and positive ANA (1:160, homogeneous) and negative myositis-associated specific antibodies. Immunology-mediated myopathies (including necrotizing autoimmune myopathies)^[19]^ and other autoimmune phenomena^[1,20–23]^ have been reported in patients with COVID-19, posing a diagnostic dilemma. After ruling out other possible diagnoses, we confirmed the presence of HT based on positive anti-TPO test results, global hypokinesia of the left ventricle, and biochemical hypothyroidism.

In electrophysiological investigations, myopathies associated with hypothyroidism often do not exhibit results consistent with irritable myopathies unless a triggering event such as myoedema or rhabdomyolysis is caused by hemodialysis, lipid-lowering medications, or vigorous exercise.^[24–33]^ Because our patient lacked any of these putative risk factors, we can deduce that COVID-19 was a physiological stressor strong enough to cause rhabdomyolysis or a polymyositis-like phenotype in patients with hypothyroidism.

Magnetic resonance imaging^[15,19]^ of the proximal lower leg muscles (given the anomalies in the electromyography readings across the vastus medialis) in conjunction with muscle biopsy^[33,34]^ would have revealed the underlying etiology of our patient’s myopathy. However, we decided not to pursue these procedures because the neurological results were normal, and the patient’s financial constraints forced us to concentrate on the most important clinical considerations. Therefore, these tests were reserved for conditions in which the CK level did not improve with levothyroxine and hydration. Since left ventricular hypokinesia was suspected to be caused by underlying ischemic cardiomyopathy, a low dose of levothyroxine was initially administered. Once hypokinesia was confirmed, mainly due to thyroid cardiomyopathy, the dose was gradually increased. The patient’s serum CK level improved significantly 14 days after starting levothyroxine administration. Even at the time of hospital discharge, on day 22 of thyroxine replacement (day 51 of hospitalization), the patient’s CK level improved tremendously but did not return to normal. We hypothesized that the patient had undetected and untreated HT caused by the SARS-CoV-2 infection.

We also reviewed the literature on the association between COVID-19 and new-onset thyroid dysfunction. Seven case reports of COVID-19-triggered HT have been published.^[6–9,35–38]^ None of the previous cases of myopathy were reported, although high levels of CK were reported in two^[8,9]^ of 7 previous reports on COVID-19-triggered HT; however, a persistent increase in CK levels was not reported. Except for 1 case,^[8]^ levothyroxine was used to treat HT and achieve euthyroidism. Except for 1 case in which HT was reportedly triggered 42 days after COVID-19,^[38]^ all other cases reported HT within 14 days of COVID-19.

Evidence from the 2002 severe acute respiratory syndrome (SARS) outbreak revealed that a small percentage of patients (6.7% of 61 patients) experienced biochemical hypothyroidism, with 3 patients exhibiting central hypothyroidism and 1 patient presenting primary hypothyroidism.^[39–41]^ In these accounts, central hypothyroidism was linked to reversible hypophysitis or a direct hypothalamic influence of the virus. However, autopsy examinations indicated damage to the follicular epithelium of the thyroid gland, which was postulated to be the origin of primary hypothyroidism.^[41]^

Although the specific association between HT and myopathy is unknown, it is assumed that the autoimmune process of HT may cause muscle inflammation, resulting in myopathy. Individuals with HT should be aware of the possibility of myopathy and report any muscular weakness or other symptoms to their healthcare providers for evaluation.^[42]^ The precise mechanisms underlying COVID-19-induced neuromuscular symptoms remain unclear. Several mechanisms have been proposed based on myopathy, myositis, and coronavirus infection pathophysiology. These mechanisms include direct viral invasion of skeletal muscles, vasculitis, or immune-mediated mechanisms without direct viral invasion, including immunological epiphenomena,^[43,44]^ or possible muscle damage due to high levels of pro-inflammatory cytokines (cytokine storm).^[1,20–24,45]^ Autoimmune processes in several patients may be associated with skeletal muscle alterations independent of the functional state of the thyroid gland.^[42]^ Given the importance of immunological dysregulation in most sequelae of COVID-19 disease, it is not unexpected that immunomodulators such as systemic glucocorticoids, hydroxychloroquine, intravenous immunoglobulin, or even tocilizumab may be required in these patients.^[15–17,19]^

While this study presents valuable insights into the rare manifestation of COVID-19, several limitations exist. First, this was a case report; therefore, generalizations cannot be made. In addition, the number of cases reported in the literature is limited, making it challenging to perform a meta-analysis or draw significant epidemiological conclusions. Furthermore, this study could not comment on the possible association between the COVID-19 variant and the severity of HT and other complications.

Despite these limitations, this case report and literature review provide essential information on the rare presentation of COVID-19, which has significant implications for clinical practice. Furthermore, this study contributes to our understanding of the mechanism of virus-triggered HT and its associated factors. As more cases have been reported in the literature, future studies may be able to explore the association between COVID-19 and HT further, leading to improved diagnosis, treatment, and treatment options.

## 4. Conclusions

This study illustrates the challenges associated with the differential diagnosis and treatment of myopathy and rhabdomyolysis in patients with COVID-19 and HT. If the clinical and biochemical abnormalities of a patient do not follow the anticipated trajectory, it is essential to consider potential alternative etiologies such as immune-mediated inflammatory myopathy or endocrinopathy-associated myopathies. Furthermore, the scarcity of data on thyroid abnormalities in COVID-19 implies that more studies are necessary to elucidate the potential relationship between thyroid dysfunction and viral infections.

## Acknowledgments

We thank the editors at www.editverse.com for their assistance in manuscript editing and proofreading.

## Author contributions

**Conceptualization:** Anindita Santosa.

**Data curation:** Lee Zheng Cong, Ti Lian Lian.

**Formal analysis:** Pande Shrikant Digambarrao, Anindita Santosa.

**Investigation:** Wong Yu Jun Eugene, Tay Tunn Lin, Anindita Santosa.

**Methodology:** Anindita Santosa.

**Project administration:** Anindita Santosa.

**Supervision:** Anindita Santosa.

**Writing – original draft:** Anindita Santosa.

**Writing – review & editing:** Lee Zheng Cong, Anindita Santosa.
